# Cultural Barriers to Women's Progression in Academic Careers: A France‐Brazil Comparison Through the Lens of the Queen Bee Phenomena

**DOI:** 10.1111/sjop.13078

**Published:** 2024-10-20

**Authors:** Catherine Esnard, Rebeca da Rocha Grangeiro

**Affiliations:** ^1^ Center for Research on Cognition and Learning (CeRCA) Université de Poitiers Poitiers France; ^2^ Laboratoire Parisien de Psychologie Sociale, Département d’Administration Économique et Sociale Université Paris Nanterre Paris France

**Keywords:** academic carrier, culture, gender, inequality, queen bee phenomena

## Abstract

Despite significant improvements, women are still underrepresented at high levels in academia. Most research on these inequalities is conducted within a specific national academic system, without taking into account its cultural roots. The aim of the present study was to analyze the extent to which the cultural context acts as a barrier on women's career progression. Specifically, we focused on psychological processes described under the metaphor of Queen Bee Phenomenon that may reflect the ways in which female academics conform to male‐gender roles encoded in androcentric social and academic culture. Two samples of women academic, one French (*N* = 73), the other Brazilian (*N* = 88), were compared through the lens of two dimension of the Queen Bee Phenomena: self‐group distancing and gender hierarchy legitimation. Brazilian women identify more with their female peer group than their French counterparts. French women are more hostile to quotas and more inclined to adhere to meritocratic discourses than their Brazilian counterparts. Both academic contexts tend to perpetuate gender inequalities, but in different ways: by maintaining gender‐stereotypical expectations in Brazil and meritocratic ideology in France. The implications for policies to promote a more egalitarian university context are discussed herein.

**Summary**

- Psychological processes described under the metaphor of Queen Bee Phenomenon may reflect the ways in which female academics conform to male‐gender roles encoded in androcentric social and academic culture.
- Both Brazilian and French academic contexts tend to perpetuate gender inequalities, but in different ways: by maintaining gender‐stereotypical expectations in Brazil and meritocratic ideology in France.

## 1 Introduction

The awareness of underrepresentation of women at high levels in academia is not recent. For almost three decades, a growing body of research has analyzed the causes of this inequality and the developing initiatives to mitigate its effects but gender disparities persist (see Llorens et al. 2021). Despite significant improvements (Thelwall 2020), women are underrepresented among the world's highly cited researchers (González‐Álvarez and Cervera‐Crespo 2019; Meho 2022) and in high‐ranking academic positions, such as university professorships and other associated positions of responsibility (Faniko, Ellemers, and Derks 2021). These disparities persist not only in the fields of science, technology, engineering and mathematics (STEM) but also in the social sciences, where most university students are women (Van Veelen and Derks 2022).

Most research on these inequalities is conducted within a specific national academic system, without taking into account its cultural roots. The aim of the present study is to analyze the extent to which the cultural context acts as a barrier on women's career progression. More specifically, one way of understanding the perpetuation of these inequalities is by examining women's attitudes through the prism of stereotypes encoded in androcentric social and academic culture (Kurchenko 2022). To achieve this, we chose to compare two academic contexts, European (French) and Brazilian, where inequalities persist despite different career paths and progress in public policies.

Several arguments support this choice. Firstly, the great majority of publications on gender inequalities in the academic context are based on research conducted on Anglo‐Saxon and European populations (Xiong et al. 2022) and, to a lesser extent, the Latin cultures of South America. Secondly, the available work highlighting characteristics specific to Brazilian culture emphasizes traditionally entrenched macho thinking that can reinforce gender biases and stereotypes. Therefore, observing the attitudes of female academics in such a context can shed light on the processes of adaptation to the dominant norm of masculinity. Thirdly, this study seeks to expand the currently limited research, on advancing female academics in Brazil.

### 1.1 Two Different Academic and Cultural Environments

In most European universities, including the French one, only two statutory positions exist (lecturer and professor). Both are attained through nationwide competitions that involve two steps: qualification and recruitment. Furthermore, the high‐status positions universities are rare and fiercely contested. Currently, in Europe, despite almost equal representation with men in the early stages of their careers, women remain underrepresented at the highest academic level, occupying around a quarter of university professor positions (26% in all European countries according to a European Commission report, “She Figures”, in November 2021). Over the past two decades, several actions to promote gender equality in higher education and research have been introduced in Europe. Considering the European Parliament Directive on the implementation of the principle of equal opportunities and equal treatment of men and women in matters of employment and occupation (2006), quotas for women on university boards and committees have become common practice. In France, the law on the transformation of the civil service (2019) introduced the obligation to draw up an action plan for professional equality between women and men in every public establishment. These plans cover four areas: pay gaps, equal access to professional categories, grades and responsibilities; work‐life balance and the fight against discrimination; violence and moral or sexual harassment and sexist behavior.

The progression in the Brazilian university system is more gradual than in European universities, with several intermediate levels. There is no need for new recruitment to reach the highest echelons; instead, advancement occurs by performing their duties conscientiously and waiting for seniority. This leads to less competition in Brazilian universities compared to their European counterparts. Whatever the case, women remain also underrepresented in the Brazilian scientific community, with their representation diminishing as career advances (de Lima et al. 2024). In Brazil, a key indicator of significant career advancement in science is achieving a Research Productivity Scholar (PQ) at the National Council for Scientific and Technological Development (CNPq). Cunha, Dimenstein, and Dantas (2021) say that despite the growing in the number of women researchers, they remain minority among the PQ/CNPq fellows, comprising only 35.6% of awardees across various PQ levels and disciplines. This disparity is more pronounced in disciplines traditionally dominated by men. In the scientific field named Engineering, Mathematics, and Earth Sciences (Ecet), women account for only 21.3% of fellows. The barriers intensify to achieve the most prestigious scientific fellowships, with women representing 26.8% of the top‐tier fellowships across all disciplines. In the Ecet domain, their presence is starkly lower, with only 0.7% holding the high‐level grants compared to 99.3% of men. This situation is unlikely to improve swiftly, as the committees responsible for setting grant criteria remain predominantly male, with 61% of members being men. Regarding management positions within Brazilian universities, they are categorized into six hierarchical levels. Andrade, Marques, and de Melo (2023) highlight a decline in female representation in positions of trust at the top three levels, with the most significant gender disparity occurring at the highest level, women accounting 19.7% of the public servants in this position. Moreover, it has been identified that the difference between Brazilian male and female scientific production is more pronounced in the 37–41 age group, when women decide to become mothers under biological clock pressure (Dellazzana‐Zanon et al. 2022). Obviously, for Brazilian academic women, the responsibility of caring for young children leads to a reduction in scientific publication for an average of four years (Ruckstadter and de Souza 2022). Given the heavy impact of parenthood in the reduction of female scientists' publications Brazilian scientists have developed a movement called Parent in Science, which has proposed the inclusion of maternity leave in the *Lattes* curriculum to take into account maternity leave when evaluating the curricula of female researchers (Carpes et al. 2022). In any case, gender equality measures are more recent, less developed than in Europe and are mainly related to motherhood.

It is clear that these public policies, regardless of the country in which they are applied, are not yet succeeding in reducing the inequalities between women and men in the academic sector. We suppose that some of the barriers to women's progression in academic careers may be due, among other factors, to the cultural pressures on women to conform to the dominant norm of masculinity prevailing in the academic context. With this mind, we referred to the Queen Bee Phenomenon (QBP).

### 1.2 The Queen Bee Phenomenon: The Necessary Adaptation to the Norm of Masculinity

There is a growing body of research that shows that in a male organization, women can likewise be involved in maintaining gender discriminations. The QBP is a metaphor used to describe psychological attitudes that a woman who has attained a leadership position is likely to adopt toward her subordinate female colleagues, thus hindering gender equality regardless of her will (Derks et al. 2011). One of the QBP characteristics is the assimilation of stereotypical male traits called agency (e.g., dominant, independent). Another attitude consists of saying things that distance oneself from women at the beginning of their careers or in junior positions who are likely to continue to confirm female gender stereotypes (e.g., women are less committed than men and less competent, especially when they have to reconcile professional and family life). Self‐group distancing is observed through the perception of the commitment, ambition, and sacrifices made in their careers as being superior to those of their subordinate colleagues. At the same time, “queen bee”(QB) women continue to identify with women of the same status who have succeeded in overcoming negative stereotypes by reaching positions of high status or leadership position. Legitimation of the gender hierarchy, the third QBP characteristic, is reflected in the attitudes of denial of the discrimination suffered by women, refusal to support public policies to combat these inequalities (e.g., affirmative action policies, that is, preferential treatment for individuals belonging to groups deemed to be disadvantaged) and support for a meritocratic ideology that promotes individuals on the basis of their merit (ability, hard work, effort, skill, intelligence, and virtue) and not of their social origin.

Often misunderstood as competition between women who have broken the glass ceiling and wish to maintain a hard‐won position, the QBP is actually a way for some women in leadership to fit into the dominant model of organizations in which adopting the norm of masculinity is the only way to be seen as qualified and evaluated more positively.

Over the last two decades, the QBP has also been identified in academic fields of higher education and research. Empirical findings show that the assimilation of male characteristics and distancing from subordinate women (doctoral students) have been observed among the senior women (professors) at universities in Italy and the Netherlands (Ellemers et al. 2004). While male and female doctoral students tend to perceive themselves as equally committed in their careers, female professors tend to perceive female doctoral students as less committed to their careers than they were in their beginning stages. Specifically, Faniko, Ellemers, and Derks (2021) highlighted the propensity of female professors to describe themselves in masculine terms as they progress, suggesting that this characteristic QBP attitude can be equated with the process of self‐group distancing. In this study, we did not consider the dimension known as “male trait assimilation,” which refers to commitment level, agency traits and career choices, because empirical findings have shown that these attitudes are more impacted by leadership positions than by gender (Faniko, Ellemers, and Derks 2016). Therefore, we chose to focus on the two characteristics that are central to the QBP: self‐group distancing and the legitimation of gender hierarchy.

Finally, by using the QBP, our study aims to contribute to the literature in this area: until now there has been work on the QBP in Europe and Brazil, but to our knowledge, no comparative study has been carried out.

### 1.3 Influence of the Brazilian Culture on Self‐Group Distancing

Although the place of women in the Brazilian labor market is increasingly important, gender inequalities persist due to the persistence of the gender roles deeply rooted in the culture of the macho Brazilian society (Vieira et al. 2017). There, work culture appears to be characterized by the valorization of hierarchy, paternalistic leadership, solidarity and the proximity between colleagues (Fang, Schaumburg, and Fjellström 2017; Stück and LeClere 2014). Even highly qualified women who are prominent in their university careers (Censon et al. 2022) report having more responsibility for domestic and caregiving activities than their partners. In general, women spend almost three times more hours a day on domestic activities than men (Loch, Torres, and Costa 2021). Moreover, in this context, women are often assigned to responsible positions by men on the basis of stereotyped female images (Colodetti and Melo 2022). Power‐related tensions are often observed in scientific careers, as identified by Barros and Mourão (2020). These authors showed how the stereotypes associated with the feminine (e.g., care, support, affectivity) influence the professional experience of women scientists. Women's internalization of the behavioral norms associated with this traditional division of roles may lead to non‐competitive attitudes in the professional sphere (Hirata and Kergoat 2007). To sum up, Brazilian university women live in an androcentric and collectivist culture which restricts women to their feminine social roles. Moreover, Brazilian academic women develop their career in an environment that is more collectivist, more cooperative, and less competitive than in France.

### 1.4 Influence of Affirmative Action Policies on Legitimization of the Gender Hierarchy

As described in the QBP literature, the legitimation of the gender hierarchy is reflected in attitudes of hostility toward affirmative action policies, such as the introduction of quotas to balance the representation of men and women and adherence to the meritocratic discourse.

According to a recent study conducted in 25 European countries between 2003 and 2018 (Forman‐Rabinovici, Mandel, and Bauer 2024), quotas are having the direct effect they were intended to have, namely increasing the representation of women on university boards. According to these authors, greater representation of women on university boards—whether achieved through quotas or not, for example, through advancement in academic careers through promotion—contributes to gender equality by providing women with symbolic forms of representation used by other women. However, the quota policy is still contested by many women who feel that they are victims of a form of positive discrimination that would deny their own qualities and competence, feel illegitimate in leadership positions when they hold them to fill quotas as analyzed in France (Grangeiro, 2021) and in Belgium (Bourabain and Verhaeghe 2022). Moreover, this parity is far from having been established among university professors, and the disparities in the professional experiences of men and women still persist. A study carried out among professors‐researchers in French universities (Drucker‐Godard et al. 2017) found that men are generally more satisfied with their work than women. Women lecturers are less committed to their universities and careers than men, but the trend is reversed or reduced when women become professors—in other words, when they have broken through the glass ceiling. In addition, when asked about their career development, French women denounce an unequal promotion system, a lack of recognition of the pedagogical tasks that are most often assigned to them to the detriment of their dedication to research and difficulties in reconciling professional and private life (Drucker‐Godard et al. 2017). In contrast, a study conducted in Brazilian universities among 703 academic men and women demonstrated that female leaders reported being more supportive of affirmative policies for women's professional development than male leaders and females without leadership positions (Grangeiro, 2022).

Numerous studies have shown that academia is marked by meritocratic norms, meaning that female and male scientists and researchers believe that their career development depends on their competence, hard work and overall merit (Fernandes et al. 2021). The rhetoric of excellence and meritocracy is produced and reproduced from the earliest stages of careers by privileged and disadvantaged groups alike (Amis, Mair, and Munir 2020). The standard of scientific excellence was designed by men and (re)produces masculine norms. Meritocratic criteria favor men over women insofar as the ideal scientist or researcher is considered as male (O'Connor 2020). In French, the qualitative study conducted by Authors (2021) indicated that female professors are prone to adopt attitudes that legitimize gender hierarchy. They support a meritocratic discourse, believing that it is up to women themselves to improve their university careers, whereas female lecturers see the university as having a major role in promoting women's careers.

### 1.5 Overview of the Study and Hypotheses

Given a less competitive posture in professional contexts and a strong commitment engagement to gender roles combined with the more collectivist Brazilian culture, we may assume (Hypothesis 1A) less self‐group distancing among Brazilian university women than among French university women and (Hypothesis 1B) less so when they are in positions of research responsibility.

Because they evolve in a cultural context that promotes gender equality and a professional mix for much longer than in Brazil, we assumed (Hypothesis 2A) that French academic women are more hostile than Brazilian academic women to political measures such as quotas and (Hypothesis 2B) more so when they are in positions of research responsibility.

We also assumed (Hypothesis 3A) that, because they evolve in a professional context more embedded in masculine and competitive norms, French academic women are more adherent to the discourse of meritocracy than Brazilian academic women and (Hypothesis 3B) more so when they are in positions of research responsibility.

## 2 Method

### 2.1 Procedures

The data were collected exclusively online, in France between April and June 2019, and in Brazil between February and April 2020. In France, the data collection was centralized at the university where participants were requested to answer a survey through the university communication system. In Brazil, data collection at a single institution generated little data, which impelled us to search for academics' institutional e‐mail addresses on the official HEI websites. That said, non‐probabilistic convenience sampling took place. Only those who agreed to participate in the study by signing the informed consent form contributed to the research. They were assured of their privacy and of the anonymity of their responses, the voluntary nature of their participation and the prerogative of quitting at any time without consequences. The average response time was 15 min.

### 2.2 Participants

As part of the larger project, the data were collected from male and female academics and administrative personnel. For the final sample, we extracted only from female academics who occupied research and/or administrative positions in French and Brazilian universities. The French sample was composed of 73 academic women and the Brazilian one of 440. To compare the two samples, we performed a random selection of 88 participants from the Brazilian sample.

A power analysis was run on G*Power with an effect size of *d* = 0.40 with the error rate set at *α* = 0.05 and the power set at *β* = 0.80. The power analysis run suggested a sample size of *N* = 156 (78 each group) to *t*‐test for two independent groups and *N* = 111 to Ancova and Anova tests. Thus, the obtained sample size of *N* = 161 (88 Brazilian academic women and 73 French academic women) was adequate to test the study hypothesis, according to the power analysis conducted on G*Power version 3.1.9.7 (Faul et al. 2007). The occupational and demographic characteristics of the participants are presented in Table 1.

**TABLE 1:** Participants characteristics.

			Brazil (*n* = 88)	France (*n* = 73)
Personal variables	Age *χ* ^*2*^ (5) = 12.511, *p* = 0.028	From 20 to 25	4	0
		From 26 to 30	4	7
		From 31 to 40	34	21
		From 41 to 50	23	16
		From 51 to 60	20	29
		Over 60	3	0
	Education *χ* ^*2*^ (1) = 11.225, *p* < 0.001	Higher education complete	6	19
		Graduate studies	82	54
	Marital status (couple) *χ* ^*2*^ (1) = 6.764, *p* = 0.052	Couple	55	56
		Not‐couple	33	17
	Children *χ* ^*2*^ (1) = 3.494, *p* = 0.062	Yes	45	48
		No	43	25
Occupation variables	Time of activity *χ* ^*2*^ (5) = 25.318, *p* < 0.001	Until 1	8	4
		From 2 to 5	25	11
		From 6 to 10	28	15
		From 11 to 15	14	6
		From 16 to 20	2	12
		Over 20	11	25
	Research responsibility *χ* ^*2*^ (1) = 67.712, *p* < 0.001	Yes	77	17
		No	11	56
	Administrative responsibility *χ* ^*2*^ (1) = 0.146, *p* = 0.702	Yes	58	46
		No	30	27

### 2.3 Measurements

For the data collection, a questionnaire was used, composed of socio‐occupational items (e.g., age, family status, time in the occupation, whether or not occupying a position of research responsibility) and a set of scales that assessed the characteristics relevant to the QBP. To examine the identification with different female subgroups, a four‐item scale (e.g., “I feel close to junior female colleagues at the beginning of their career”) was used (Faniko et al. 2012). To assess the legitimization of the gender hierarchy, we used two items (e.g., “During my career in the university, women and men received equal career support”) of the scales of discrimination denial (Derks et al. 2011), five items (e.g., “In universities, people who do their job well ought to rise to the top”) of adherence to meritocratic principles (Davey et al. 1999) and three items (e.g., “I am in favor of applying quota hiring policy”) of quota support (Faniko et al. 2012).

The authors translated and adapted the scales to the French and Brazilian contexts. Reverse translation was then undertaken by a professional English teacher, and few differences were identified between the reverse translation and the original scale items. Subsequently, a pre‐test was performed with four university professors in order to verify any errors or non‐understanding that would indicate a need for small adaptations. Finally, a seven‐point Likert scale was used ranging from 1 (I totally disagree) to 7 (I totally agree).

Psychometric and reliability tests of the scales were conducted, through which Cronbach *α*, variance, KMO, and Bartlett's Sphericity test were tested. Table 1 details these tests. The scales show significant and reliable results, and the only necessary change was the exclusion of one item in the identification scale (see Table 2).

**TABLE 2:** Psychometric and scale reliability results.

	Scale	Number of items	France				Brazil			
			Cronbach *α*	Variance (%)	KMO	Bartlett's Sphericity	Cronbach *α*	Variance (%)	KMO	Bartlett's Sphericity
Identification with same gender colleague	At the bottom of the hierarchy	2	0.34	59.56	0.50	*χ* ^*2*^ = 2.62, 1 gL, ns	0.34	37.65	0.50	*χ* ^*2*^ = 22.53, 1 gL, *p* < 0.001
	At the top the hierarchy	2	0.86	88.04	0.50	*χ* ^*2*^ = 60.98, 1 gL, *p* < 0.001	0.59	70.89	0.50	*χ* ^*2*^ = 94.51, 1 gL, *p* < 0.001
Legitimization	Discrimination denial	2	0.79	82.59	0.50	*χ* ^*2*^ = 38.99, 1 gL, *p* < 0.001	0.80	83.64	0.50	*χ* ^*2*^ = 296.86, 1 gL, *p* < 0.001
	Quota support	3	0.88	81.41	0.66	*χ* ^*2*^ = 171.38, 3 gL, *p* < 0.001	0.90	83.16	0.69	*χ* ^*2*^ = 1101.05, 3 gL, *p* < 0.001
	Adherence to meritocratic principles	5	0.75	50.88	0.74	*χ* ^*2*^ = 83.69, 10 gL, *p* < 0.001	0.69	46.40	0.73	*χ* ^*2*^ = 453.76, 10 gL, *p* < 0.001

## 3 Results

The participants' demographic features, such as age, marital state, and level of education, had no effect on the QBP attitudes. We hypothesized that Brazilian university women in positions of responsibility declared less self‐group distancing than French university women in positions of responsibility (Hypothesis 1A) and less so when they were in positions of research responsibility (Hypothesis 1B). To test Hypothesis 1A, we conducted a Student *t*‐test. The Brazilian academic women (*M* = 5.24, *SD* = 0.84) reported themselves as more identified with their female colleagues than the French academic women (*M* = 4.26, *SD* = 1.13). The difference between the groups was significant (*t*(159) = 6.316, *p* < 0.001). Therefore, we confirmed Hypothesis 1A.

To test Hypothesis 1B, we performed an analysis of covariance (ANCOVA). The covariate, origin was significantly related to the self‐group distancing, *F*(1,158) = 15.84, *p* < 0.01. Nevertheless, there was no significant effect of having a responsibility‐based research post on self‐group distancing after controlling for the effect of origin, *F*(1,158) = 1.667, *p* = 0.19. Therefore, we rejected Hypothesis 1B.

Even though they evolve in a cultural context that promotes gender equality and professional mix, we hypothesized that French academic women are more hostile to policy measures that promote gender equality than Brazilian academic women (Hypothesis 2A), and more so when they are in positions of research responsibility (Hypothesis 2B). To test Hypothesis 2A, we conducted a Student *t*‐test. The French academic women (*M* = 3.94, *SD* = 1.73) declared less support for quotas than the Brazilian academic women (*M* = 4.52, *SD* = 1.79). The difference between the groups was significant (*t*(159) = 2.047, *p* = 0.04). Therefore, we confirmed Hypothesis 2A.

To test Hypothesis 2B, we performed an ANCOVA. The covariate, origin, was not significantly related to the support of quotas, *F*(1,158) = 1.26, *p* = 0.263. Also, there was no significant effect of having a responsibility‐based research post on the support of quotas after controlling for the effect of origin, *F*(1,158) = 0.446, *p* = 0.505. Therefore, we rejected Hypothesis 2B.

Moreover, we hypothesized that, because they evolve in a professional context more embedded in masculine and competitive norms, French academic women are more adherent to the discourse of meritocracy than Brazilian academic women (Hypothesis 3A) and more so when they are in positions of research responsibility (Hypothesis 3B). To test Hypothesis 3A, we conducted a Student *t*‐test. The French academic women (*M* = 5.26, *SD* = 1.02) declared more adherence to meritocratic discourse than did the Brazilian academic women (*M* = 4.35, *SD* = 1.02). The difference between the groups was significant (*t*(158,384) = −4.917, *p* < 0.001). Therefore, we confirmed Hypothesis 3A.

To test Hypothesis 3B, we performed an ANCOVA. The covariate, origin, was significantly related to the adhesion to meritocracy, *F*(1,158) = 29.13, *p* < 0.01. There was also a significant effect of having a responsibility‐based research post on the adhesion to meritocracy after controlling for the effect of origin, *F*(1,158) = 6.67, *p* = 0.011. Therefore, we confirmed Hypothesis 3B.

## 4 Discussion

The objective of this study was to analyze how cultural barriers may affect women in the development of their academic careers. Precisely, the analysis focused on the psychological processes—self‐group distancing and gender hierarchy legitimation—that may reflect the way in which women academics conform to the cultural characteristics structuring their relationships to their professional environment. Two samples of academic women in positions of responsibility were compared—one French, the other Brazilian.

As proposed, the Brazilian academic women reported less self‐group distancing than the French academic women; this effect was observed regardless of their positions in terms of research responsibility. This result probably reflects the fact that, in a culture strongly marked by patriarchy (Vieira et al. 2017; Waight et al. 2022), while concomitantly pursuing an academic career to a high level, Brazilian women academics conform more to stereotypical female role expectations than do French women academics. The identification between women seems to reinforce and strengthen a sense of collectivity that is a stronger structural feature in the Brazilian than in the French academic context. In a society historically marked by patriarchal norms, women, even those with high professional status, are often more responsible for family caring than their romantic partners. The promotion of inclusive work contexts which improves women's leadership performance (Bodla et al. 2023), while respecting self‐identification within the female group, is a relatively new demand in Brazil and needs to be introduced gradually.

Active gender equality policies are more developed and have existed for a longer time in French than in Brazilian universities. However, there has been a “backlash” in Europe against so‐called positive discrimination (known in the USA as “affirmative action”). As we assumed, French female academics are more hostile than Brazilian female academics to gender quota policies and this effect is observed regardless of their position in terms of research responsibility. This may be due to the fact that Brazilian university women have not yet had any experience of the results of their country's gender equality policies in practice. For example, apart from a few isolated cases, the period of maternity is not taken into account in the calculation of their scientific productivity, as required by the country's Lattes Curriculum Guidelines. On the contrary, given the real‐life consequences of the active gender equality policies and possible problems in their daily work due to the imposition of quotas, French women academics may be rendered resistant to positive discrimination. Furthermore, we may assume that the more pronounced sense of collectivity characteristic of Brazilian culture and the minimal self‐group distancing among the Brazilian university women identified in this study may result in greater support for affirmative policies among Brazilian than among French women academics.

At last, as we hypothesized, the French women academics reported being more adherent to meritocratic discourse than their Brazilian counterparts. Moreover, holding positions of research responsibility increases adherence to the discourse of meritocracy—this more for French than for Brazilian academic women. This seems to reflect the fact that, while androcentric culture limits Brazilian women's ambitions for academic advancement, it also keeps them out of a historically male professional context with rules and ideals created by and advantaging men more than women, such as excellence, brilliance, and complete dedication. On the contrary, and even more so when they reach research responsibilities, French women academics immersed in a competitive environment tend to reproduce the meritocratic discourse. Indeed, the more competitive and embedded in male norms they are, the more they must believe that career advancement is based on gender‐neutral criteria. This is because it is threatening for women with career ambitions to assume that their gender would put them at a disadvantage in competition with male candidates (Fernandes et al. 2021).

By examining the queen bee phenomenon (QBP) in France and Brazil, this study propounds an original comparison between two countries with marked cultural differences. Thereby, it highlights the cultural determinants that may affect the QBP in an academic context; on the one hand, patriarchal and collectivist culture reduces self‐group distancing among women and is likely to strengthen the cohesion and solidarity among women, while a more meritocratic setting helps to legitimize gender hierarchy. Our results consolidate knowledge on QBP, in particular the importance of self‐group distancing (Faniko, Ellemers, and Derks 2021) and the need to consider meritocratic ideology, especially in an academic context. Meritocratic principles suggest that the university constitutes a fair and just context, meaning that the abilities demonstrated and efforts made will be rewarded regardless of gender, even though the role expectations conducive to progressing and succeeding in an academic career are male. Paradoxically, women's adherence to meritocratic principles is an obstacle to active gender equality policies in the academic context (Grangeiro, 2024). Consequently, this study should contribute to the reflection on how gender inequalities and discriminatory practices are experienced, perceived, and addressed in academic settings. To take this reflection further, we need to consider the implicit processes that drive gender discrimination, and in particular, the ways in which women sometimes come to justify the inequalities of which they are victims. Two conceptual frameworks that have given rise to empirical studies in a single‐sector context can be drawn upon here: the theory of system justification (Jost and Banaji 1994), which allows us to understand how and why gender stereotypes that are seen as complementary ensure the stability of the social systems in place and the theory of core values (Schwartz et al. 2012), which allows us to analyze the potential inadequacy of women to the dominant values in the organization (Aelenei et al. 2020).

In terms of practical implications, this study should contribute to reflection on how gender inequalities and discriminatory practices are experienced and perceived and addressed in academic settings. The examination of two distinct academic cultures should facilitate an exchange of experiences regarding gender diversity practices. For example, gender diversity managers in Brazilian universities may be able to anticipate some of the consequences of quota implementation in European universities. Specifically, the representation of women in management positions obtained through quotas by no means guarantees the absence of gender discrimination. This study supports the observations and analysis of Deschamps (2023) on French universities, where the government decided in 2015 to impose a quota of women on university recruitment and promotion committees. However, the presence of women on these committees does not necessarily promote the actual recruitment of women and may even be detrimental to their careers. In fact, the author notes a surprising correlation that could suggest a queen bee effect: the higher the proportion of women on a committee, the lower the ranking of female candidates, regardless of the quality of their applications. In other words, quotas for high‐ranking positions may, in the medium and long term, help to transform male culture, provided that women leaders effectively implement measures designed to promote equality. However, it bears mentioning that not all women in positions of power develop QBP attitudes; on the contrary, some help to increase the proportion of women in high‐status positions as soon as they reach a position of responsibility (Arvate, Galilea, and Todescat 2018). It likewise bears mentioning that the recognition and reward of the communal practices existing in Brazilian universities, especially with regard to maternity leave, could potentially provide inspiration for communal gender diversity practices in French universities. In addition, the valuing of communal, teamwork‐based practices may, over time, weaken agentic gender norms for development and success in research careers.

## 5 Limitations And Future Research Directions

This study has some limitations that can be addressed in future work. It is still exploratory based on only a few elements that distinguish the two cultural contexts: the more traditional male‐dominated and collectivist culture in Brazil than in France and the more cooperative academic environment in Brazil versus the more competitive in France; its results will need to be confirmed by a systematic cultural comparison. Notably, given the apparent importance of adherence to meritocracy, it will be necessary to combine the data collection with the assessment of the ideological postures that guide women academics in the conduct of academic projects. It will also be useful to more clearly identify how a real or perceived competitive academic context conditions the ideological postures and professional strategies of women academics. Moreover, this study focused on limited samples of university women. In future work, the data collection should be extended to different university contexts (e.g., institutions of varying size, prestige, and endowment resources). Finally, future work will need to integrate the analysis of the obstacles that male academics may face, the objective being to foster a more inclusive academic setting.

## 6 Conclusion

An inclusive and egalitarian university means not assuming that there are two types of leadership, one male and the other female. To achieve this, public policies in favor of gender diversity must be implemented at two levels. At the individual level, this means recognizing the psychological processes, rooted in values and stereotypes that contribute to the perpetuation of inequalities, on the part of both women and men. At a structural level, it is essential to rethink increased competitiveness and the pursuit of excellence, as they condition behaviors and attitudes that leave little room for diverse leadership styles, regardless of gender role expectations.

## Author Contributions

All authors designed the study, collected and analyzed the data, discussed the results and wrote the manuscript.

## Ethics Statement

All procedures performed in this study were in accordance with the ethical standards of the institutional research committee at [anonymized for review] and with the 1964 Helsinki Declaration and its later amendments or comparable ethical standards.

## Consent

Informed consent was obtained from all individual adult participants included in this study.

## Conflicts of Interest

The authors declare no conflicts of interest.

## Data Availability

The data that support the findings of this study are openly available in figshare at https://doi.org/10.6084/m9.figshare.21901461.

## References

[sjop13078-bib-0001] Aelenei, C. , D. Martinot , A. Sicard , and C. Darnon . 2020. “When an Academic Culture Based on Self‐Enhancement Values Undermines Female students' Sense of Belonging, Self‐Efficacy, and Academic Choices.” Journal of Social Psychology 160, no. 3: 373–389. 10.1080/00224545.2019.1675576. 31600124

[sjop13078-bib-0002] Amis, J. M. , J. Mair , and K. A. Munir . 2020. “The Organizational Reproduction of Inequality.” Academy of Management Annals 14, no. 1: 195–230. 10.5465/annals.2017.0033.

[sjop13078-bib-0003] Andrade, A. L. , A. C. R. Marques , and M. R. de Melo . 2023. “Female Career Barriers in the Public Service.” Revista Pensamento Contemporâneo em Administração 17, no. 4: 140–159. 10.12712/rpca.v17i4.59923.

[sjop13078-bib-0004] Arvate, P. R. , G. W. Galilea , and I. Todescat . 2018. “The Queen Bee: A Myth? The Effect of Top‐Level Female Leadership on Subordinate Females.” Leadership Quarterly 29, no. 5: 533–548. 10.1016/j.leaqua.2018.03.002.

[sjop13078-bib-0045] Barros, S. C. D. V. , and L. Mourão . 2020. “Professional Career of Women Scientists in the Light of Gender Stereotypes.” Psicologia em Estudo 25: 1–16. 10.4025/psicolestud.v25i0.46325.

[sjop13078-bib-0005] Bodla, A. A. , Y. Li , A. Ali , and A. S. Hernandez Bark . 2023. “Female leaders' Social Network Structures and Managerial Performance: The Moderating Effects of Promotional Orientation and Climate for Inclusion.” Scandinavian Journal of Psychology 64, no. 2: 160–170. 10.1111/sjop.12875. 36200591

[sjop13078-bib-0006] Bourabain, D. , and P. P. Verhaeghe . 2022. “Shiny on the Outside, Rotten on the Inside? Perceptions of Female Early Career Researchers on Diversity Policies in Higher Education Institutions.” Higher Education Policy 35, no. 2: 542–560. 10.1057/s41307-021-00226-0.

[sjop13078-bib-0007] Capes. Coordenação de Aperfeiçoamento de Pessoal de Nível Superior . 2019. “Dados Abertos Capes.” Brasília. https://www.gov.br/mcti/pt‐br/composicao/rede‐mcti/conselho‐nacional‐de‐desenvolvimento‐cientifico‐e‐tecnologico.

[sjop13078-bib-0008] Carpes, P. B. M. , A. R. P. Abreu , F. Staniscuaski , M. A. Souza , M. J. Campagnole‐Santos , and M. C. Irigoyen . 2022. “Actions Developed by the Brazilian Physiological Society to Promote women's Participation in Science.” Advances in Physiology Education 43: 199–206. 10.1152/advan.00216.2018. 30998104

[sjop13078-bib-0009] Censon, D. , C. U. F. D. Reis , J. Medaglia , and M. S. M. Nakatani . 2022. “The Trajectories of Women in Tourism Teaching and Research.” Revista Brasileira de Pesquisa em Turismo 16: e2468. 10.7784/rbtur.v16.2468.

[sjop13078-bib-0010] Colodetti, A. P. D. O. A. , and M. C. D. O. L. Melo . 2022. “Gender Relations in the Brazilian Socioeconomic and Cultural Context: A Study With Female Drivers of Urban Mobility Apps.” Cadernos EBAPE. BR 19: 872–886. 10.1590/1679-39512020141.

[sjop13078-bib-0011] Cunha, R. , M. Dimenstein , and C. Dantas . 2021. “Gender Inequalities by Field of Knowledge in Brazilian Science: An Overview of the PQ/CNPq Female Researchers.” Saúde em Debate 45, no. spe1: 83–97. 10.1590/0103-11042021E107.

[sjop13078-bib-0012] Davey, L. M. , D. R. Bobocel , L. S. S. Hing , and M. P. Zanna . 1999. “Preference for the Merit Principle Scale: An Individual Difference Measure of Distributive Justice Preferences.” Social Justice Research 12: 223–240. 10.1023/A:1022148418210.

[sjop13078-bib-0043] de Lima, J. P. , S. P. Nova , and E. de Oliveira Vendramin . 2024. “Sexist Academic Socialization and Feminist Resistance: (de)constructing Women's (dis)placement in Brazilian Accounting Academia.” Critical Perspectives on Accounting 99: 102600. 10.1016/j.cpa.2023.102600.

[sjop13078-bib-0013] Dellazzana‐Zanon, L. L. , A. L. Dellazzana , M. P. de Sousa , and L. dos Santos Souza . 2022. “Gender (In)equality in the Academic Career: The Impact of Motherhood.” Revista Brasileira de Pós‐Graduação 18, no. especial: 1–16.

[sjop13078-bib-0014] Deschamps, P. 2023. “Gender Quotas in Hiring Committees: A Boon or a Bane for Women?” Management Science. 10.1287/mnsc.2022.01637.

[sjop13078-bib-0015] Derks, B. , N. Ellemers , C. Van Laar , and K. Groot . 2011. “Do Sexist Organizational Cultures Create the Queen Bee?” British Journal of Social Psychology 50: 519–535. 10.1348/014466610X525280. 21884548

[sjop13078-bib-0016] Drucker‐Godard, C. , T. Fouque , M. Gollety , and A. Le Flanchec . 2017. “Enseignant‐chercheur au féminin: La place des femmes dans les universités.” Recherches en Sciences de Gestion 118, no. 1: 125–145. 10.3917/UHVJ.118.0125.

[sjop13078-bib-0017] Ellemers, N. , H. V. D. Heuvel , D. De Gilder , A. Maass , and A. Bonvini . 2004. “The Underrepresentation of Women in Science: Differential Commitment or the Queen Bee Syndrome?” British Journal of Social Psychology 43: 315–338. 10.1348/0144666042037999. 15479533

[sjop13078-bib-0018] EU Directive . 2006. “EU Directive 2006/54/EC of the European Parliament and of the Council on the Implementation of the Principle of Equal Opportunities and Equal Treatment of Men and Women in Matters of Employment and Occupation, 5 July 2006.” Official Journal of the European Union L 204: 23–36. https://eur‐lex.europa.eu/eli/dir/2006/54/oj.

[sjop13078-bib-0019] European Commission, Directorate‐General for Research and Innovation . 2021. She Figures 2021: Gender in Research and Innovation: Statistics and Indicators. Luxembourg: Publications Office of the European Union. 10.2777/06090.

[sjop13078-bib-0020] Fang, T. , J. Schaumburg , and D. Fjellström . 2017. “International Business Negotiations in Brazil.” Journal of Business & Industrial Marketing 32, no. 4: 591–605. 10.1108/JBIM-11-2016-0257.

[sjop13078-bib-0021] Faniko, K. , N. Ellemers , and B. Derks . 2016. “Queen Bees and Alpha Males: Are Successful Women More Competitive Than Successful Men?” European Journal of Social Psychology 46: 903–913. 10.1002/ejsp.2198.

[sjop13078-bib-0022] Faniko, K. , N. Ellemers , and B. Derks . 2021. “The Queen Bee Phenomenon in Academia 15 Years After: Does It Still Exist, and if So, Why?” British Journal of Social Psychology 60, no. 2: 383–399. 10.1111/bjso.12408. 32696985 PMC8246980

[sjop13078-bib-0023] Faniko, K. , F. Lorenzi‐Cioldi , F. Buschini , and A. Chatard . 2012. “The Influence of Education on Attitudes Toward Affirmative Action: The Role of the policy's Strength.” Journal of Applied Social Psychology 42: 387–413. 10.1111/j.1559-1816.2011.00892.x.

[sjop13078-bib-0024] Faul, F. , E. Erdfelder , A.‐G. Lang , and A. Buchner . 2007. “G*Power 3: A Flexible Statistical Power Analysis Program for the Social, Behavioral, and Biomedical Sciences.” Behavior Research Methods 39: 175–191. 10.3758/BF03193146. 17695343

[sjop13078-bib-0025] Fernandes, C. , M. L. Lourenço , S. Frohlich , D. E. D. Silva , and F. O. Kai . 2021. “Women in Politics: Emotions and Challenges in Complex Institutional Dynamics.” Cadernos EBAPE. BR 18: 1071–1081. 10.1590/1679-395120200006.

[sjop13078-bib-0026] Forman‐Rabinovici, A. , H. Mandel , and A. Bauer . 2024. “Legislating Gender Equality in Academia: Direct and Indirect Effects of State‐Mandated Gender Quota Policies in European Academia.” Studies in Higher Education 49: 1134–1150. 10.1080/03075079.2023.2260402.

[sjop13078-bib-0027] González‐Álvarez, J. , and T. Cervera‐Crespo . 2019. “Contemporary Psychology and Women: A Gender Analysis of the Scientific Production.” International Journal of Psychology 54, no. 1: 135–143. 10.1002/ijop.12433. 28503775

[sjop13078-bib-0046] Grangeiro, R. , and C. Esnard . 2021. “Le phénomène de la Reine des Abeilles: quelles particularités à l'Université?” Cadernos de Pesquisa 51: e07516. 10.1590/198053147516.

[sjop13078-bib-0047] Grangeiro, R. , M. Gomes Neto , and C. Esnard . 2022. “Women in Leadership Positions in Universities: Are They Really Queen Bees?” Management Research Review 46, no. 5: 739–754. 10.1108/MRR-03-2021-0239.

[sjop13078-bib-0048] Grangeiro, R. , M. B. Gomes Neto , L. E. N. Silva , and C. Esnard . 2024. “The Triggers and Consequences of the Queen Bee Phenomenon: A Systematic Literature Review and Integrative Framework.” Scandinavian Journal of Psychology 65, no. 1: 86–97. 10.1111/sjop.12957. 37599206

[sjop13078-bib-0028] Hirata, H. , and D. Kergoat . 2007. “Novas configurações da divisão sexual do trabalho.” Cadernos de Pesquisa 37: 595–609. 10.1590/S0100-15742007000300005.

[sjop13078-bib-0030] Jost, J. T. , and M. R. Banaji . 1994. “The Role of Stereotyping in System‐Justification and the Production of False Consciousness.” British Journal of Social Psychology 33, no. 1: 1–27.

[sjop13078-bib-0031] Kurchenko, L. 2022. “Invisible Barriers, Undeclared Wars: Subtle Resistances to Women's Leadership in Academia.” In Policy and Practice Challenges for Equality in Education, 1–23. Hershey, PA: IGI Global. 10.4018/978-1-7998-7379-2.ch001.

[sjop13078-bib-0032] Llorens, A. , A. Tzovara , L. Bellier , et al. 2021. “Gender Bias in Academia: A Lifetime Problem That Needs Solutions.” Neuron 109, no. 13: 2047–2074. 10.1016/j.neuron.2021.06.002. 34237278 PMC8553227

[sjop13078-bib-0033] Loch, R. M. B. , K. B. V. Torres , and C. R. Costa . 2021. “Woman, Wife and Mother in Science and Technology.” Revista Estudos Feministas 29, no. 1: e61470. 10.1590/1806-9584-2021v29n161470.

[sjop13078-bib-0034] Meho, L. I. 2022. “Gender Gap Among Highly Cited Researchers, 2014–2021.” Quantitative Science Studies 3, no. 4: 1003–1023. 10.1162/qss_a_00218.

[sjop13078-bib-0035] O'Connor, P. 2020. “Why Is It So Difficult to Reduce Gender Inequality in Male‐Dominated Higher Educational Organizations? A Feminist Institutional Perspective.” Interdisciplinary Science Reviews 45, no. 2: 207–228. 10.1080/03080188.2020.1737903.

[sjop13078-bib-0036] Ruckstadter, V. C. M. , and R. E. de Souza . 2022. “Affirmative Actions for Mothers as a Mechanism of Isonomy and Strengthening the Public University: A Report From the Experience at Universidade Estadual Do Norte Do Paraná (UENP).” Vivências 18, no. 35: 121–132. 10.31512/vivencias.v18i35.487.

[sjop13078-bib-0037] Schwartz, S. H. , J. Cieciuch , M. Vecchione , et al. 2012. “Refining the Theory of Basic Individual Values.” Journal of Personality and Social Psychology 103, no. 4: 663–688. 10.1037/a0029393. 22823292

[sjop13078-bib-0044] Stück, J. , and M. J. LeClere . 2014. “The Brasileiro and the Yankee: A Cross‐Cultural Comparison of Brazilian and American Managers in Brazil.” Exchange 3, no. 1: 68–81. https://ssrn.com/abstract=2526398.

[sjop13078-bib-0038] Thelwall, M. 2020. “Author Gender Differences in Psychology Citation Impact 1996–2018.” International Journal of Psychology 55, no. 4: 684–694. 31782157 10.1002/ijop.12633

[sjop13078-bib-0039] Van Veelen, R. , and B. Derks . 2022. “Equal Representation Does Not Mean Equal Opportunity: Women Academics Perceive a Thicker Glass Ceiling in Social and Behavioral Fields Than in the Natural Sciences and Economics.” Frontiers in Psychology 13: 1–19. 10.3389/fpsyg.2022.790211. PMC8966382 35369222

[sjop13078-bib-0040] Vieira, A. , A. D. P. Carrieri , P. R. R. Monteiro , and F. F. Roquete . 2017. “Gender Differences and Professional Identities in Health and Engineering.” Brazilian Administration Review 14, no. 1: 1–21. 10.1590/1807-7692bar2017160082.

[sjop13078-bib-0041] Waight, C. L. , T. N. Kjerfve , A. Kite , and B. Smith . 2022. “Connecting and Relating in Brazil: Implications of Remote Work.” Human Resource Development International 25, no. 2: 231–253. 10.1080/13678868.2022.2048435.

[sjop13078-bib-0042] Xiong, A. , S. Xia , Q. Wang , et al. 2022. “Queen Bees: How Is Female managers' Happiness Determined?” Frontiers in Psychology 13: 741576. 10.3389/fpsyg.2022.741576. 35250703 PMC8888411

